# The association between hepatitis C virus infection and renal cell cancer, prostate cancer, and bladder cancer: a systematic review and meta-analysis

**DOI:** 10.1038/s41598-021-90404-2

**Published:** 2021-05-25

**Authors:** Yucheng Ma, Zhongli Huang, Zhongyu Jian, Xin Wei

**Affiliations:** Department of Urology, Institute of Urology, Laboratory of Reconstructive Urology), West China Hospital, Sichuan University, No. 37 Guo Xue Xiang, Chengdu, 610041 Sichuan People’s Republic of China

**Keywords:** Cancer, Cancer epidemiology

## Abstract

To update the current evidence on whether hepatitis C virus (HCV) infection represents a possible risk factor for renal cell cancer (RCC), prostate cancer (PCa), and bladder cancer (BC). We searched the literature on Pubmed, Web of Science, and Embases before April 2021. A systematic review and meta-analysis were performed. Finally, we extracted 12 studies based on the eligible criteria. Across 11 studies for HCV and RCC, the incorporated RR was 1.28 (95% CI 1.05–1.55), which meant that participants with HCV infection were associated with higher RCC risk. The pooled RR in hazard ratio (HR) subgroup (HR 1.59, 95% CI 1.22–2.08), cohort studies subgroup (RR 1.47, 95% CI 1.18–1.82), and North America subgroup (RR 1.71, 95% CI 1.40–2.09) detected a stronger association between HCV and RCC risk. Although an inverse association was seen for PCa (RR 0.75, 95% CI 0.54–1.03) across seven studies, it was not statistically significant (*P* = 0.075). There was no significant association between HCV and BC with an incorporated RR of 0.92 (95% CI, 0.82–1.03) across five studies. Our study demonstrated that HCV infection was significantly associated with increased RCC risk. There appeared to be an inverse association for HCV in PCa risk but not statistically significant. No significant association was found between HCV and BC risk. Prospective, large-scale, and well-designed cohort studies are required to validate the association between HCV and RCC, and to investigate the role of HCV on PCa.

## Introduction

Urologic cancers such as renal cell cancer (RCC), prostate cancer (PCa), and bladder cancer (BC) are most common diagnosed cancers in humans^1^. Both non-modifiable and environmental risk factors are identified associated with these cancers. However, the role of hepatitis C virus (HCV) infection on these cancers is still controversial.

HCV infection is a public health problem. It was estimated that approximately 180 million people infected worldwide^2^. HCV infection has been found to be involved in both hepatic diseases and a variety of extra-hepatic diseases^3^. Although the mechanism of how HCV could affect these cancers was not clearly comprehended, the association between HCV and RCC, PCa, and BC has been extended researched.

A meta-analysis published in 2016 reported an association in HCV infection for RCC risk^4^. However, the state of the two studies used in meta-analysis was estimated and crude, which was one of the limitations. In addition, more studies were published afterward, and the results were still inconclusive. To conclude a more robust estimation of their associations, we updated the current evidence on whether HCV infection represents a possible risk factor for RCC. There was no meta-analysis focusing on the association between HCV and PCa or BC to our knowledge. Although HCV infection rates are on the decline globally, there are still a large number of people living with HCV infection in absolute terms. If the association between HCV and urological tumors can be established, regular urological tumor screening in this population may significantly extend their survival time.

In light of these controversial roles of HCV on RCC, PCa, and BC risk, the purpose of the present meta-analysis is to explore whether HCV represents a possible risk factor for RCC, PCa, and BC by taking all available studies meeting our inclusion criteria into consideration.

## Materials and methods

To perform this study, we followed the guide of Preferred Reporting Items for Systematic Reviews and Meta-Analyses (PRISMA) guidelines^5^.

### Search strategy

Two independent reviewers (YM and ZJ) searched literature on Pubmed, Web of Science, and Embases before April 2021. Relevant studies restricted to the English language were identified according to the eligibility criteria as follows. The Search terms include Hepatitis C Virus, HCV, Prostatic Neoplasms, Prostate Cancer, Renal Cell Cancer, Renal Cancer, Kidney Neoplasm, Kidney Cancer, Renal Neoplasm, Renal Cell Cancer, Bladder Neoplasm, Bladder Cancer. Negotiated between 2 reviewers or consulted with a third author were used to resolve the disagreements.

### Eligible criteria

Eligible criteria are described as follows. (1) Population: Patients with RCC, PCa, or BC; (2) Exposure: HCV infection; (3) Comparison: participants control; (4) Outcomes: diagnosis any kind of these three cancers; (5) Study: all study designs. Studies meeting the eligibility criteria listed above and the any kind of relative risk (RR) estimates (standardized incidence ratio [SIR], hazard ratio [HR] or odds ratios [OR]) and their 95% confidence interval (CI) could be directly extracted were included in our meta-analysis. Besides, review, meta-analysis, letters, abstracts, case reports, meeting comments, editorials, and congress reports were excluded from our study.

### Data extraction and quality assessment

Two independent reviewers (YM and ZJ) assessed study quality, followed by extracting data from the individual study. Discussion and reevaluation of the methodology would be adopted to resolve the disagreements. Information including the name of the first author, study location, study period, study design, publication year, sample size, measurement of cancers (RCC, PCa, and BC), and the RR (95% CIs) for each category of cancers. The checklist was used to evaluate the quality used was the Newcastle–Ottawa Scale^6^ (NOS) tool. The total score of each study was nine, and a score of more than six was classified into the high-quality group.

### Statistical analyses

Effect measures (SIR, OR, and HR) with 95% CI was extracted from the eligible study, and a final combined RR and its 95% CI was obtained by the inverse-variances method. The I^2^ statistics with Q test was used to assess heterogeneity among included studies^7^. For pooled RR, the level of statistical heterogeneity was used to choose a primary statistical model. When heterogeneity was significant (I^2^ > 50% or *P * < 0.1), a random effect model would be used. Otherwise, the fixed-effect model was applied when substantial heterogeneity was not observed. Next, subgroup analyses were performed to identify which clinical factor might contribute to the potential source of heterogeneity. Then, we performed sensitivity analysis by omitting individual studies one by one. Finally, both Begg and Egger test was conducted to identify publication bias. A two-sided *P* < 0.05 in our study indicated a significant statistical difference. Statistical analyses were performed with Stata version 14 software (Stata Corporation, College Station, TX, USA).

## Results

A total of 644 studies using the search criteria were identified. Finally, we extracted 12 studies^8–19^ based on the eligible criteria (Supplementary Fig. S1). The characteristics were demonstrated in Table 1. We found seven cohort studies and five case–control studies that investigated the role of HCV for RCC. There were seven studies reporting the association between HCV and PCa, of which 5 were cohort studies. For studies exploring the role of HCV on BC, 3 were cohort studies, and 2 were case–control studies. The detailed NOS score of each study belongs to cohort studies, and the case–control studies design was shown in Supplementary Tables S1 and S2, respectively.

**Table 1:** Characteristics of studies included in the systematic review and meta-analysis.

Study	Publication year	Study design	Study location	Study period	Total numbers	HCV patient number	Cancer numbers	HCV confirmation	Cancer confirmation	Outcomes RR;95%CI	NOS score
Nyberg^8^	2019	CS	California, USA	2008–2012	5,332,903	35,712	9751 in PCa	ICD-9 code confirmation and/or a positive HCV RNA test	Confirmed by KPSC-National Cancer Institute Surveillance, Epidemiology and End Results (KPSC-NCI SEER) affiliated cancer registry	[A] HR 1.71; 1.29, 2.25 [B] HR 1.45; 1.24, 1.69	7
Liu^9^	2019	CCS	China	2008–2016	34,505	189	1287 in RCC	positive for anti-HCV	Confirmed by pathological and histological examination	[A] OR 0.71; 0.23, 2.25	5
Liu^10^	2017	CS	Sweden	1990–2010	29,271	29,271	24 in RCC; 35 in BC; 93 in PCa	positive for anti-HCV and HCV-RNA analysis by real-time PCR	Confirmed by nationwide Swedish Cancer Registry	[A] SIR 1.42; 0.91, 2.12 [B] SIR 0.73; 0.59, 0.90 [C] SIR 1.20; 0.84, 1.67	6
Lin^11^	2017	CCS	Taiwan	2000–2011	53,241	1034	17,747 in RCC	positive for anti-HCV	Confirmed by Registry for Catastrophic Illness Patient (RCIP) database	[A] OR 1.24; 1.07, 1.44	7
Mahale^12^	2017	CCS	United Kingdom	1993–2011	1,823,538	12,107	35,960 in RCC; 90,360 in BC; 283,367 in PCa	NR	Confirmed by Surveillance, Epidemiology, and End Results (SEER) datasets	[A] OR 0.89; 0.76, 1.04 [B] OR 0.84; 0.48, 1.46 [C] OR 0.88; 0.77, 1.00	8
Kamiza^13^	2016	CCS	Taiwan	1995–2014	44,150	35,320	1065 in RCC; 1324 in BC; 1820 in PCa	NR	Confirmed by Longitudinal Health Insurance Database 2000 and The National Health Insurance Research Database	[A] HR 0.98; 0.49, 1.93 [B] HR 1.55; 1.04, 2.32 [C] HR 1.20; 0.75, 1.90	6
Gonzalez^14^	2015	CCS	Michigan, USA	2011–2013	240	9	140 in RCC	positive HCV RNA test	Confirmed by pathological and histological examination	[A] OR 24.20; 2.4, N/A	N/A
Allison^15^	2015	CS	USA	2006–2010	12,126	12,126	17 in RCC; 46 in PCa	positive HCV RNA test and International Classification of Diseases confirmed HCV infection	Confirmed by Chronic Hepatitis Cohort Study (CHeCS), Surveillance, Epidemiology, and End Results (SEER) cancer registry datasts and Multiple Causes of Death (MCOD) datasets	[A] SIR 1.7; 1.1, 2.2 [C] SIR 0.6; 0.5, 0.7	8
Hofmann^16^	2011	CS	Sweden	1990–2006	43,000	43,000	29 in RCC	NR	Confirmed by national Cancer, Cause of Death and Hospital Discharge registers datasets	[A] SIR 1.2; 0.8, 1.7	6
Omland^17^	2010	CS	Denmark	1994–2003	4204	4204	4 in RCC; 2 in BC; 2 in PCa	NR	Confirmed by the Danish National Hospital Registry (DNHR) datasets	[A] SIR 3.6; 0.98, 9.22 [B] SIR 1.02; 0.12, 3.7 [C] SIR 0.92; 0.11, 3.31	7
Gordon^18^	2010	CS	USA	1997–2006	72,487	3057	194 in RCC	positive HCV RNA test	Confirmed by pathological and histological examination	[A] HR 1.77; 1.05, 2.98	8
Amin^19^	2006	CS	Sydney	1990–2002	75,834	75,834	18 in RCC; 11 in BC; 32 in PCa	positive for anti-HCV and HCV-RNA analysis	Confirmed by NSW Central Cancer Registry (CCR) datasets	[A] SIR 0.9; 0.6, 0.4 [B] SIR 0.4; 0.3, 0.6 [C] SIR 0.7; 0.4, 1.2	8

[A] = kidney cancer; [B] = bladder cancer; [C] = prostate cancer; N/A = not available; HCV = hepatitis C; RCC = renal cell cancer; BC = bladder cancer; PCa = prostate cancer; RR = relative risk; HR = hazard ratio; OR = odds ratio; SIR = standardized incidence ratio; NOS = Newcastle–Ottawa Scale; CS = cohort study; CCS = case–control study; N/A = not available; not evaluated.

### HCV and RCC

**Figure 1:**
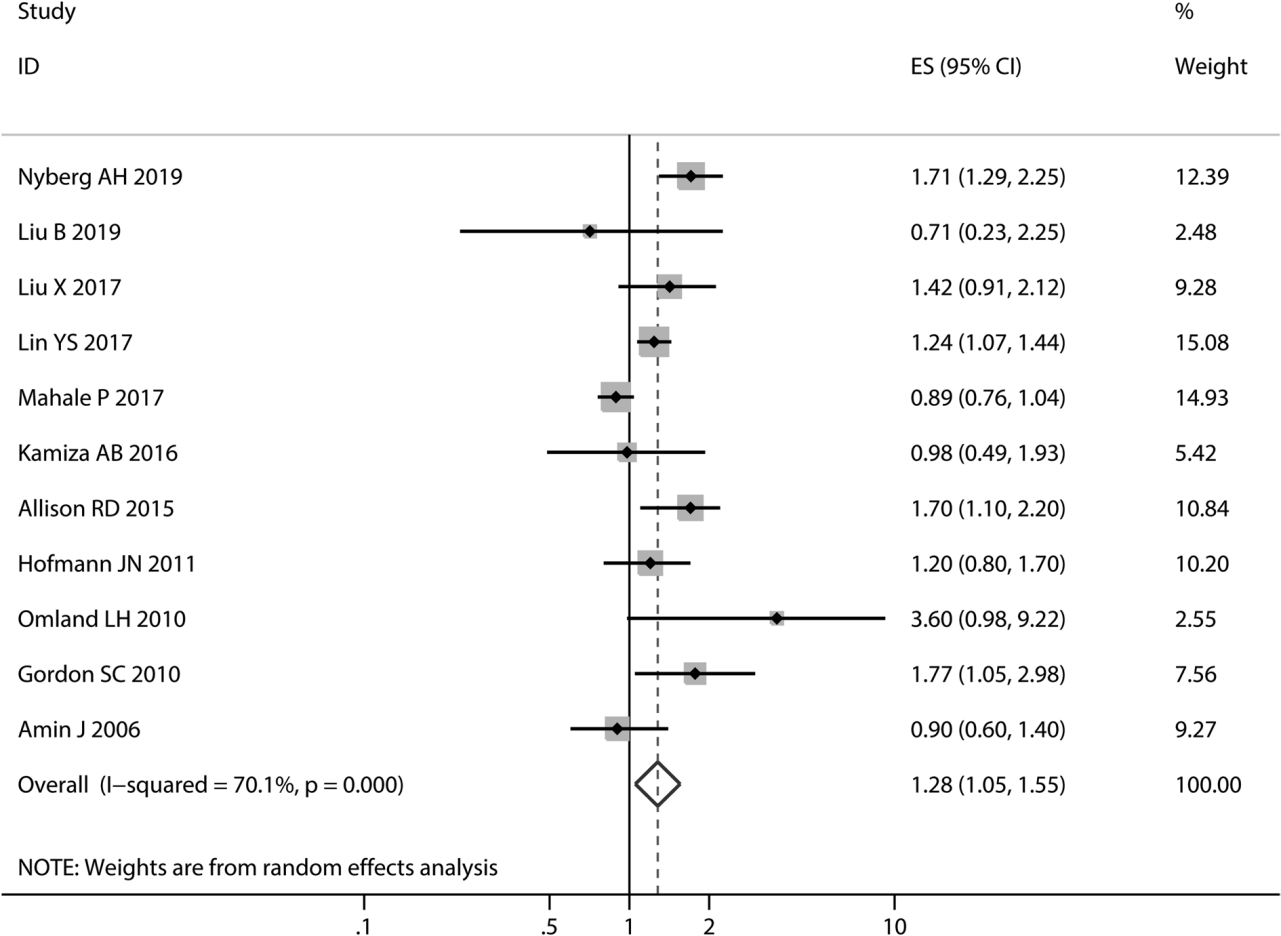
Forest plots of relative risk of studies investigating the association between hepatitis C virus infection and renal cell cancer. Random effects models were used for the primary meta-analysis.

For HCV and RCC, 11 studies^8–13,15–19^ had sufficient data for meta-analysis. As shown in Fig. 1, the summary of RR obtained by the random-effects model (I^2^ = 70.1%; *P * < 0.001) was 1.28 (95% CI 1.05–1.55), which meant participants with HCV infection had a significantly higher risk of RCC.

We further assessed the clinical factors that might potential contribute to the heterogeneity (Table 2). The pooled RR in HR subgroup (HR 1.59, 95% CI 1.22–2.08; I^2^ = 13.5%, *P* = 0.315), cohort studies subgroup (RR 1.47, 95% CI 1.18–1.82; I^2^ = 45.6%, *P* = 0.088), and USA subgroup (RR 1.71, 95% CI 1.40–2.09; I^2^ = 0, *P* = 0.991) detected a stronger association between HCV and RCC risk.

**Table 2:** Subgroup analysis of association between HCV and RCC risk.

Category of variables	Studies, n	Heterogeneity		RR (95%CI)	*P * value for difference
		I^2^, %	*P * value		
***RCC***	***11***	***70.1***	** < *0.001***	***1.28(1.05–1.55)***	***0.013***
Type of RR					
HR	3	13.5	0.315	1.59(1.22–2.08)	***0.001***
OR	3	79.0	0.009	1.03(0.76–1.39)	0.866
SIR	5	53.4	0.072	1.37(1.01–1.84)	***0.040***
Study design					
Cohort study	7	45.6	0.088	1.47(1.18–1.82)	** < 0.001**
Case–control study	4	68.7	0.023	1.02(0.79–1.33)	0.857
Geographic area					
North America	3	0	0.991	1.71(1.40–2.09)	** < *0.001***
Asia	3	0	0.521	1.22(1.05–1.40)	***0.008***
Europe	3	71.9	0.014	1.23(0.85–1.79)	0.267
Australia	1	–	–	0.90(0.59–1.37)	0.626
Publication year					
After 2012	7	76.6	< 0.001	1.26(0.99–1.59)	0.057
Before 2012	4	59.9	0.058	1.37(0.90–2.07)	0.142
NOS score					
≥ 7	7	81.0	< 0.001	1.34(1.04–1.73)	***0.024***
≤ 6	4	0	0.625	1.21(0.94–1.56)	0.144
≤ 6	2	0	1.000	1.20(0.91–1.58)	0.196

**Figure 2:**
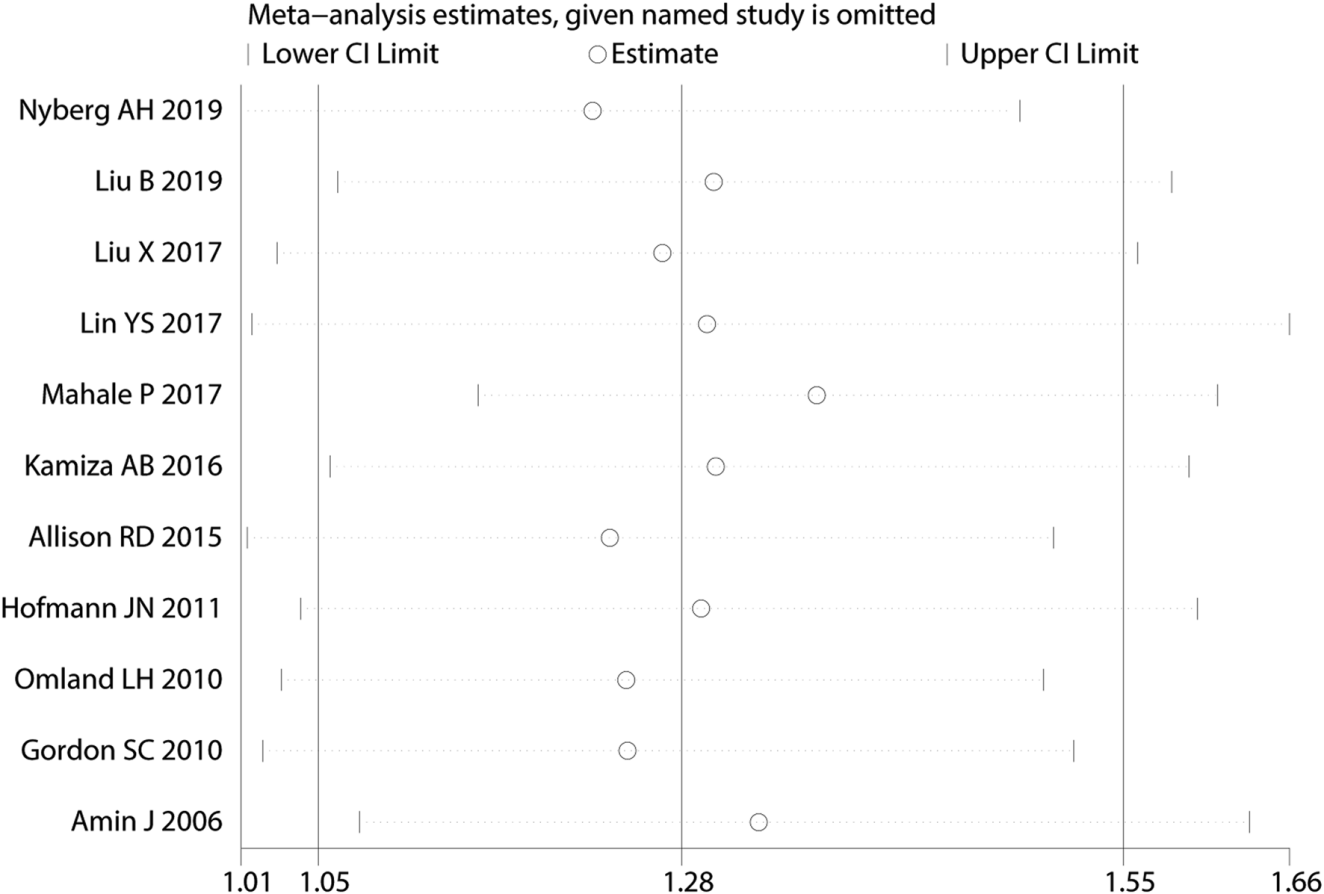
Sensitivity analyses by omitting individual study yielded nonsignificant RR change.

HCV = hepatitis C; RCC = renal cell carcinoma; BC = bladder cancer; PCa = prostate cancer; RR = relative risk; HR = hazard ratio; OR = odds ratio; SIR = standardized incidence ratio; NOS = Newcastle–Ottawa Scale;

Sensitivity analyses were shown in Fig. 2. By omitting individual study yielded nonsignificant RR change ranging from 1.22 (95% CI 1.01–1.49) to 1.36 (95% CI 1.15–1.61). Both Begg rank correlation test (*P* >|z|= 0.938) (Supplementary Fig. S2) and Egger linear regression (*P* >|t|= 0.172) demonstrated that there was no significant publication bias among these studies.

### HCV and PCa

Across a total of 7 studies^8,10,12–15,17,19^ which examined the role of HCV for PCa, there was no significant association between HCV and PCa was found, with a pooled RR of 0.75 (95% CI 0.54–1.03). Between-study heterogeneity was significant based on the I^2^ = 93% and Q statistic (P < 0.001) and, so a random-effects model was applied (Supplementary Fig. S3).

Analyses in case–control studies subgroup (RR 1.71, 95% CI 1.40–2.09; I^2^ = 0, *P* = 0.991), Europe subgroup (RR 1.71, 95% CI 1.40–2.09; I^2^ = 0, *P* = 0.991), studies published before 2012 subgroup (RR 1.71, 95% CI 1.40–2.09; I^2^ = 0, *P* = 0.991), and subgroup with lower NOS score (RR 1.71, 95% CI 1.40–2.09; I^2^ = 0, *P* = 0.991) found a significant inverse association with very low heterogeneity. However, we must treat it with caution because of the limited number and relatively low methodological quality studies included in these subgroups. Similarly, a significant inverse association observed in SIR subgroup (RR 0.58, 95% CI 0.45–0.76) should be interpreted carefully because of the substantial heterogeneity (I^2^ = 66.1, *P* = 0.031) (Table 3).

**Table 3:** Subgroup analysis of association between PCa risk.

Category of variables	Studies, n	Heterogeneity		RR (95%CI)	*P * value for difference
		I^2^, %	*P * value		
***PCa***	***7***	***93.0***	** < *0.001***	***0.75(0.54–1.03)***	***0.075***
Type of RR					
HR	2	70.6	0.065	1.18(0.71–1.98)	0.529
OR	1	–	–	0.73(0.66–0.81)	< 0.001
SIR	4	66.1	0.031	0.58(0.45–0.76)	** < *0.001***
Study design					
Cohort study	5	95.1	< 0.001	0.74(0.44–1.22)	0.231
Case–control study	2	0	0.627	0.73(0.66–0.82)	** < *0.001***
Geographic area					
North America	2	98.2	< 0.001	0.93(0.39–2.21)	0.873
Asia	1	–	–	0.84(0.48–1.47)	0.539
Europe	3	0	0.930	0.73(0.66–0.81)	** < *0.001***
Australia	1	–	–	0.40(0.28–0.57)	< 0.001
Publication year					
After 2012	5	94.3	< 0.001	0.83(0.59–1.16)	0.274
Before 2012	2	9.1	0.294	0.43(0.26–0.71)	***0.001***
NOS score					
≥ 7	5	95.3	< 0.001	0.73(0.48–1.13)	0.160
≤ 6	2	0	0.644	0.74(0.61–0.91)	***0.003***

HCV = hepatitis C; RCC = renal cell carcinoma; BC = bladder cancer; PCa = prostate cancer; RR = relative risk; HR = hazard ratio; OR = odds ratio; SIR = standardized incidence ratio; NOS = Newcastle–Ottawa Scale;

One individual study affected the overall results, a significant inverse association was found between HCV and PCa (RR 0.65, 95% CI 0.54–0.77) (Supplementary Fig. S4). However, substantial heterogeneity was still high (I^2^ = 64.2, *P* = 0.016). Publication bias was not significant based on Begg (*P* >|z|= 0.293) (Supplementary Fig. S5). and Egger (*P* >|t|= 0.882) tests.

### HCV and BC

The pooled RR enrolling five studies^10,12,13,17,19^ was 0.92 (95% CI, 0.82–1.03), representing no significant association between HCV infection and BC risk. Heterogeneity was not significant based on the I^2^ = 19.2% and Q statistic (*P* = 0.293), so a fixed-effects model was used (Supplementary Fig. S6).

Subgroup analyses revealed that the pooled RR in higher NOS score subgroup was statistic significant (RR 0.87, 95% CI 0.77–0.99; I^2^ = 0, *P* = 0.728) (Table 4). Influence analysis revealed that none study had a greater impact on the pooled RR (Supplementary Fig. S7). No significant publication bias was found according to the Begg (*P* >|z|= 1.000) (Supplementary Fig. S8). and Egger (*P* >|t|= 0.536) tests.

**Table 4:** Subgroup analysis of association between BC risk.

Category of variables	Studies, n	Heterogeneity		RR (95%CI)	*P * value for difference
		I^2^, %	*P * value		
***BC***	***5***	***19.2***	***0.293***	***0.92(0.82–1.03)***	***0.154***
Type of RR					
HR	1	–	–	1.03(0.77–1.37)	0.851
OR	1	–	–	0.88(0.77–1.37)	0.055
SIR	3	25.2	0.262	1.37(1.01–1.84)	0.442
Study design					
Cohort study	3	25.2	0.262	1.37(1.01–1.84)	0.442
Case–control study	2	36.9	0.208	0.90(0.79–1.02)	0.102
Geographic area					
North America	0	–	–	–	**–**
Asia	1	–	–	1.20(0.75–1.91)	0.442
Europe	3	26.9	0.255	0.92(0.81–1.03)	0.154
Australia	1	–	–	0.70(0.40–1.21)	0.203
Publication year					
After 2012	3	49.4	0.138	0.93(0.83–1.05)	0.238
Before 2012	2	0	0.765	0.72(0.43–1.21)	0.215
NOS score					
≥ 7	3	0	0.728	0.87(0.77–0.99)	***0.031***
≤ 6	2	0	1.000	1.20(0.91–1.58)	0.196

HCV = hepatitis C; RCC = renal cell carcinoma; BC = bladder cancer; PCa = prostate cancer; RR = relative risk; HR = hazard ratio; OR = odds ratio; SIR = standardized incidence ratio; NOS = Newcastle–Ottawa Scale;

## Discussion

This meta-analysis had several important findings with respect to the role of HCV infection on RCC, PCa, and BC. First, we found a significantly increased risk of association between HCV and RCC (Fig. 1). This association was detected stronger in the HR subgroup, cohort study subgroup, and North America subgroup accompany with the obviously decreased heterogeneity (Table 2). As we omitted each study in sensitivity analyses (Fig. 2) but nevertheless a nonsignificant trend appeared. Second, although an inverse association was seen for PCa (RR 0.75) in meta-analysis enrolling seven studies, it was not statistically significant (*P* = 0.075). In subsequent subgroup analyses.

HCV infection was associated with a significantly decreased risk of PCa in the case–control studies subgroup, Europe subgroup, studies published before the 2012 subgroup, and lower NOS score subgroup (Table 2). However, we must interpret it carefully because of the limited number and relatively low methodological quality studies included in these subgroups. Third, regardless of sensitivity analyses, pooled RR remained nonsignificant between HCV infection and BC risk.

RCC incidence has increased over the past two decades, the role of HCV infection on RCC was still inconclusive. A recent meta-analysis^4^ found that HCV infection had an increased risk of RCC, but the state of two studies used in meta-analysis were estimated and crude. Therefore, our study provided a more precise estimation of the role of HCV for RCC. The association was most strong (RR = 1.71) in USA subgroup analyses seemed to be intriguing. As we know, an estimated 4.1 million individuals in the USA have been exposed to HCV^20^. Besides, RCC incidence has increased, particularly among African Americans^21^. Therefore, it seems to be reasonable to consider screening newly diagnosed RCC for HCV infection in the USA.

Although the mechanism by which HCV increased the risk of RCC is not completely understood, the most important explanation is that HCV-associated chronic kidney disease (CKD) might play an important role. HCV infection was found to be associated with developing CKD and end-stage renal disease^22^. In addition, several other hypotheses have been proposed. First, the HCV virus core protein and the NY-REN-54 protein contribute to the influence of HCV on RCC^23^. Second, serine protease inhibitor Kazal (SPIK) protein can inhibit serine protease-related apoptosis, and HCV was found that could increase the expression of SPIK^24^. Third, cytotoxic T-cell-dependent apoptosis plays a pilot role in host immunity and normal tissue. HCV can disturb this process and lead to renal oncogenesis^25^. In conclusion, our evidence supported the promoting role of HCV on RCC, and the mechanism needs to be elucidated in future studies.

For HCV and PCa, there was no significant association according to our overall meta-analysis (Table 3). Up to now, few explanations about this issue was reported. Amin et al.^19^ and Lee et al.^26^ were the first to report an association between HCV and PCa incidence, HCV and PCa mortality, respectively. However, both did not discuss the potential reasons. Krystyna et al.^27^ reported that HCV infection increased the risk of PCa because they believed more serologic testing would be done if their physician suspected cancer. On the contrary, Mahale et al.^12^ reported an inverse association between HCV and PCa. They concluded that those HCV-infected individuals often come from lower socioeconomic status groups, so the rate of prostate cancer screening is relatively low. As we know, controlling confounding factors well was methodologically challenged in studies which tried to conclude the modifiable risk factors for cancers because the carcinogenesis of cancers was multifactorial. In fact, there will be more meaningful to explore the HCV based on PCa grade or stage, because many of PCa were not clinically relevant. In summary, we did not find that HCV was associated with PCa. Although some subgroup analyses showed an inverse association, further prospective, large-scale, long follow-up, and well-controlled confounders cohort studies are needed to investigate this association.

At the design stage of this study, we assumed that HCV infection might be positively associated with the development of bladder cancer as there were some reported molecular biological processes that might be involved^28^. However, there was no significant association found between them according to this meta-analysis results (Table 4). As an important urine storage organ of the human body, the bladder has been in contact with a large amount of stationary and non-flowing urine for a long time, so it has also become a vital target organ for many carcinogens^29^. Several other viruses have also been shown to be significantly associated with bladder cancer, and the possible mechanism is that the genetic material of the virus, such as RNA, travels through the blood into the urine and affects the transitional cells of the bladder^30–32^. The reasons for the failure to find a significant association between HCV and bladder cancer in this study may be varied, and further validation of this conclusion may require epidemiological investigation with larger sample size, mainly covering the high-risk population of HCV infection.

Several potential limitations should not be ignored. First, the heterogeneity in meta-analysis for RCC and PCa was high. On the one hand, random-effects models were applied for making results to be conservative. On the other hand, we explored the potential clinical factors which might contribute to heterogeneity by subgroup analysis methods. Nevertheless, the residual heterogeneity could not be interpreted sufficiently. Second, the small number of studies included in the meta-analysis for BC was a limitation. Third, this study was limited by pooling a few low NOS score (< 7) studies. Lastly, the absence of stratification on grade or stage in these cancers (RCC, PCa, and BC) was a limitation that prevented us from better investigating the clinical significance of HCV on these cancers.

## Conclusion

With or without sensitivity analyses, the summary estimates from our meta-analysis demonstrated that HCV infection was significantly associated with increased RCC risk, especially enrolling studies in USA locations. Although an inverse association was seen for HCV and PCa risk, it was not statistically significant. There was no significant association between HCV infection and BC risk. Prospective, large-scale, and well-designed cohort studies are needed to validate the association between HCV and RCC and to investigate the role of HCV on PCa.

## Supplementary Information

Supplementary Information.
