# Supplementary material for: The association between hepatitis C virus infection and renal cell cancer, prostate cancer, and bladder cancer: a systematic review and meta-analysis
Source: Sci Rep. 2021 May 25;11:10833. doi: 10.1038/s41598-021-90404-2 (PMC8149817; doi:10.1038/s41598-021-90404-2)
Supplement: Supplementary file 1 — Supplementary Information. [file 41598_2021_90404_MOESM1_ESM.pdf]

Title Page

Title: The Association Between Hepatitis C Virus Infection and Renal Cell Cancer, Prostate Cancer, and Bladder Cancer: A Systematic Review and Meta-Analysis

Authors:

Yucheng Ma, yuchengma88@163.com

Zhongli Huang, huangzhongli1234@gmail.com

Zhongyu Jian, 1178590069@qq.com

Xin Wei*. weixinscu@scu.edu.cn

Yucheng Ma, Zhongli Huang and Zhongyu Jian contribute equally

Affiliations of authors: Department of Urology, Institute of Urology（Laboratory of Reconstructive Urology）, West China Hospital, Sichuan University, Chengdu, Sichuan, P.R.C;

Address: Department of Urology, West China Hospital, Sichuan University, No. 37 Guo Xue Xiang, Chengdu, Sichuan, 610041, P.R. China.

***Correspondence author:** Xin Wei, Department of Urology, Institute of Urology（Laboratory of Reconstructive Urology）, West China Hospital, Sichuan University, Chengdu, 610041, China. Email: weixinscu@scu.edu.cn， Phone: +86 18982081721

Supplementary Figure S1 Preferred Reporting Items for Systematic Reviews and meta-analysis flowchart.

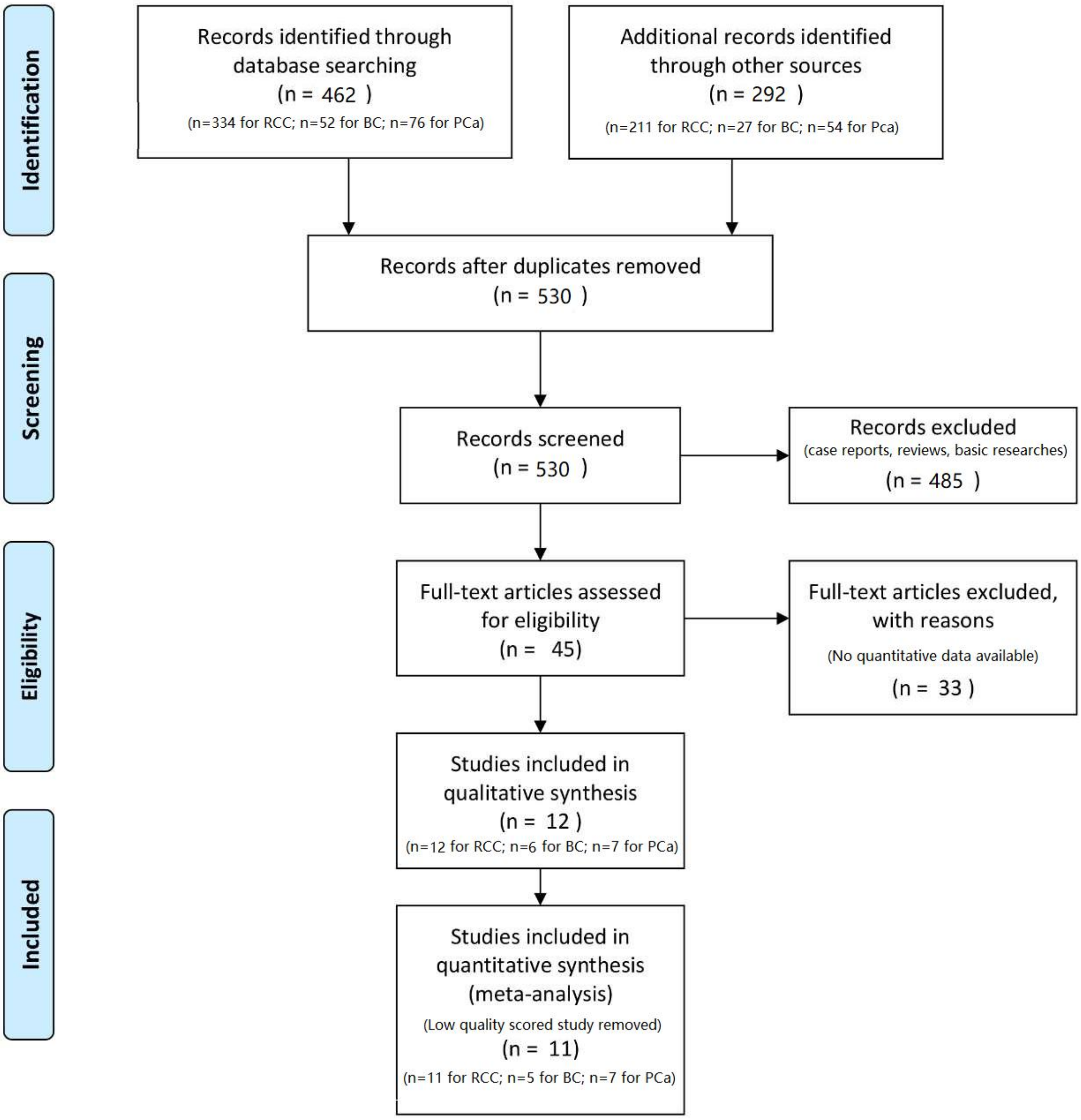

Supplementary Figure S2 Begg test to detect publication bias in meta-analysis investigating the association between hepatitis C virus infection and renal cell cancer.

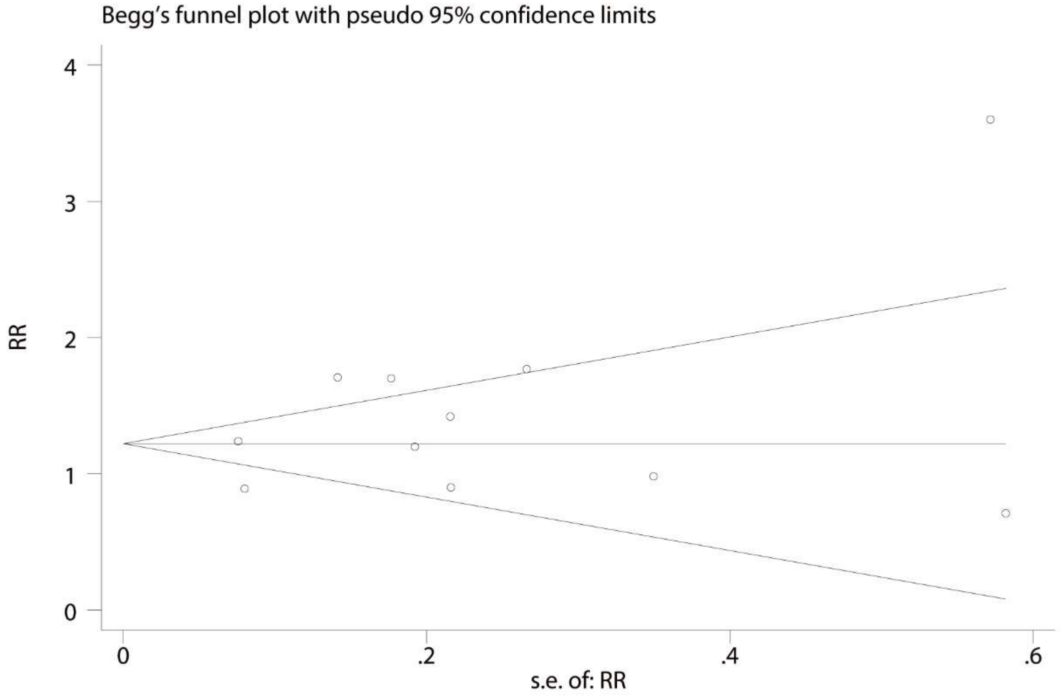

Supplementary Figure S3 Forest plots of relative risk of studies investigating the association between hepatitis C virus infection and prostate cancer. Random effects models were used for the primary meta-analysis.

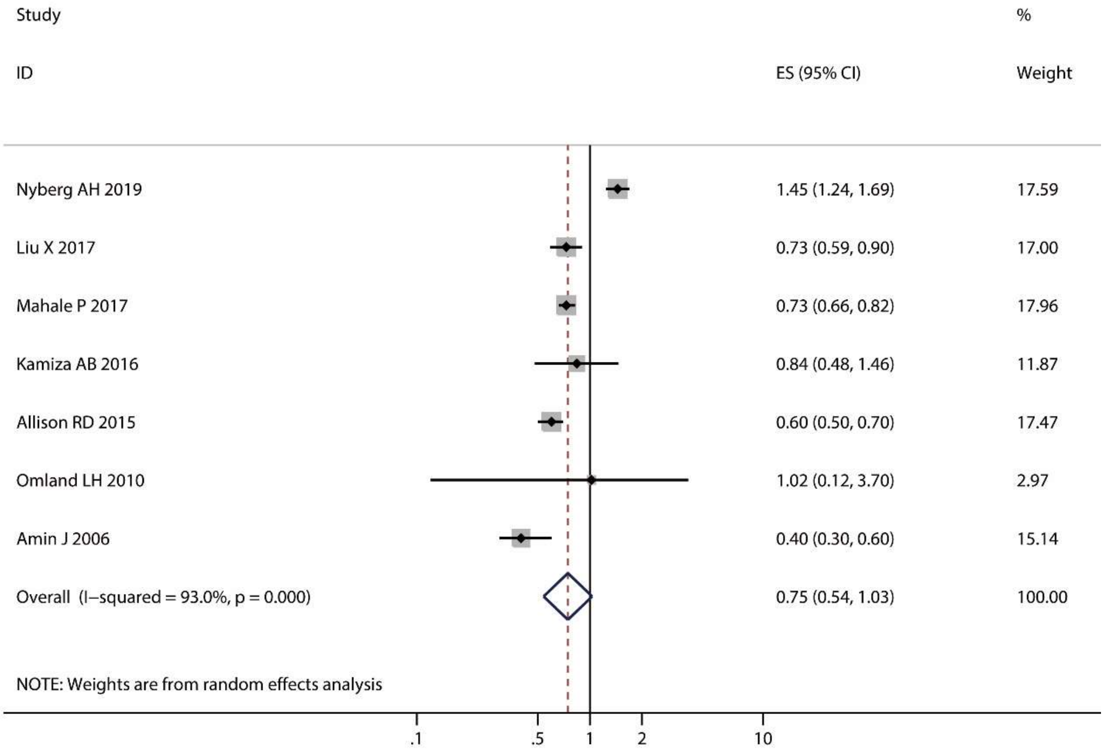

Supplementary Figure S4 Sensitivity analyses by omitting individual study in meta-analysis investigating the association between hepatitis C virus infection and prostate cancer.

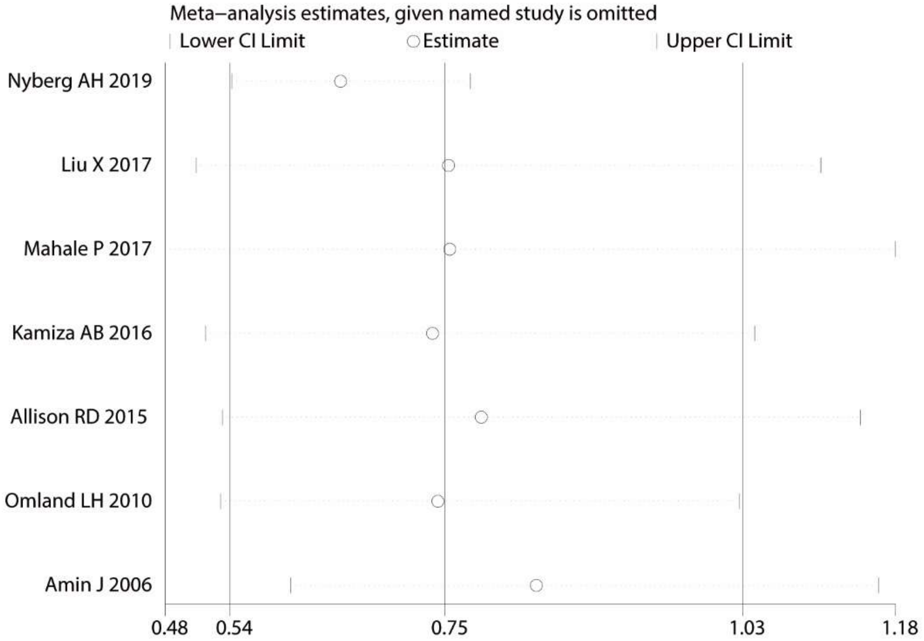

Supplementary Figure S5 Begg test to detect publication bias in meta-analysis investigating the association between hepatitis C virus infection and prostate cancer.

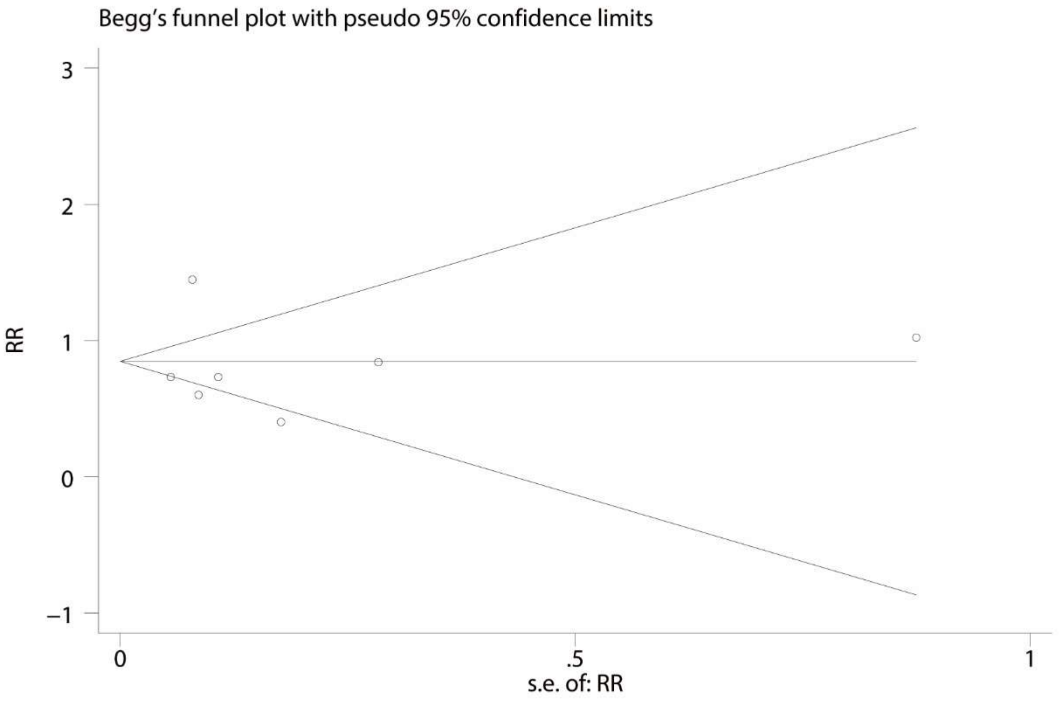

Supplementary Figure S6 Forest plots of relative risk of studies investigating the association between hepatitis C virus infection and bladder cancer. Fixed effects models were used for the primary meta-analysis.

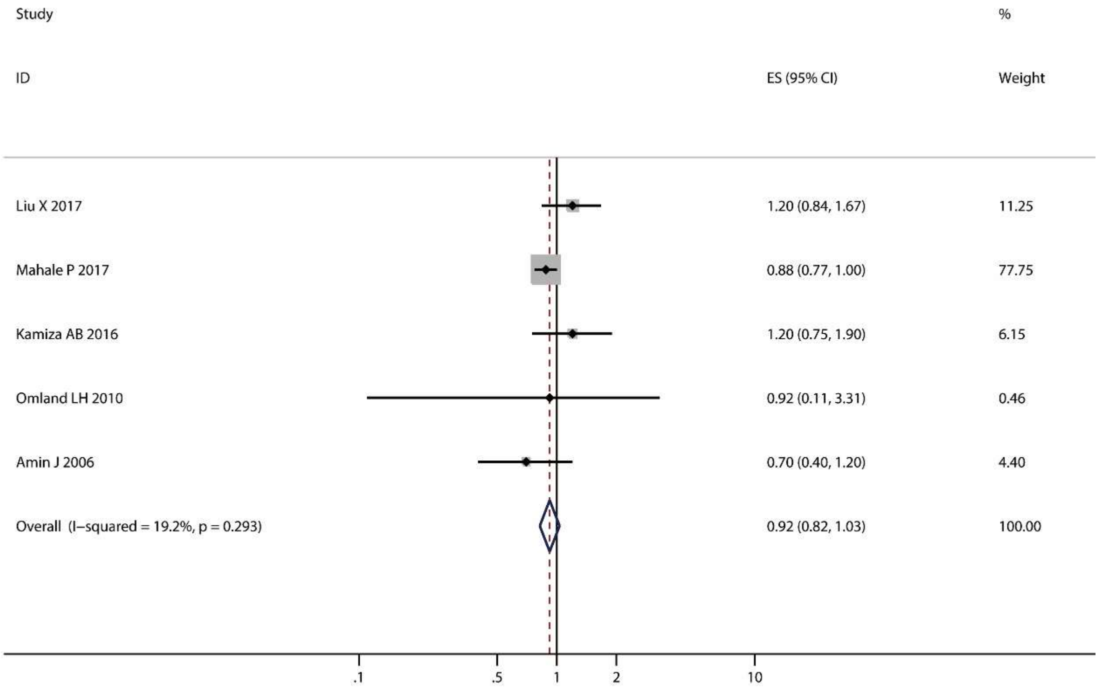

Supplementary Figure S7 Sensitivity analyses by omitting individual study in meta-analysis investigating the association between hepatitis C virus infection and bladder cancer.

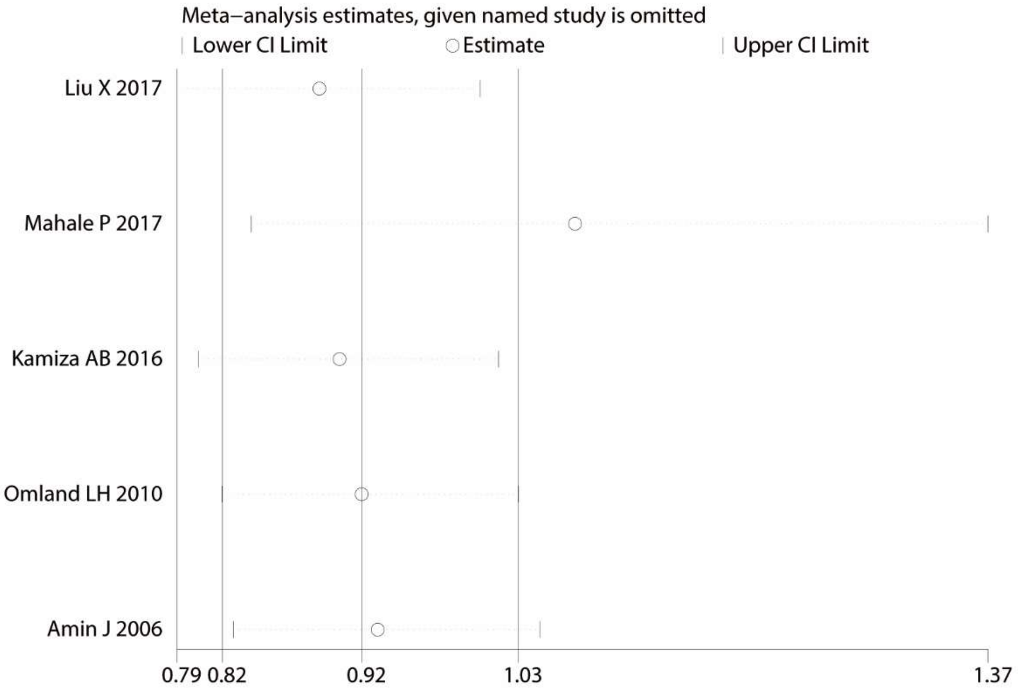

Supplementary Figure S8 Begg test to detect publication bias in meta-analysis investigating the association between hepatitis C virus infection and bladder cancer.

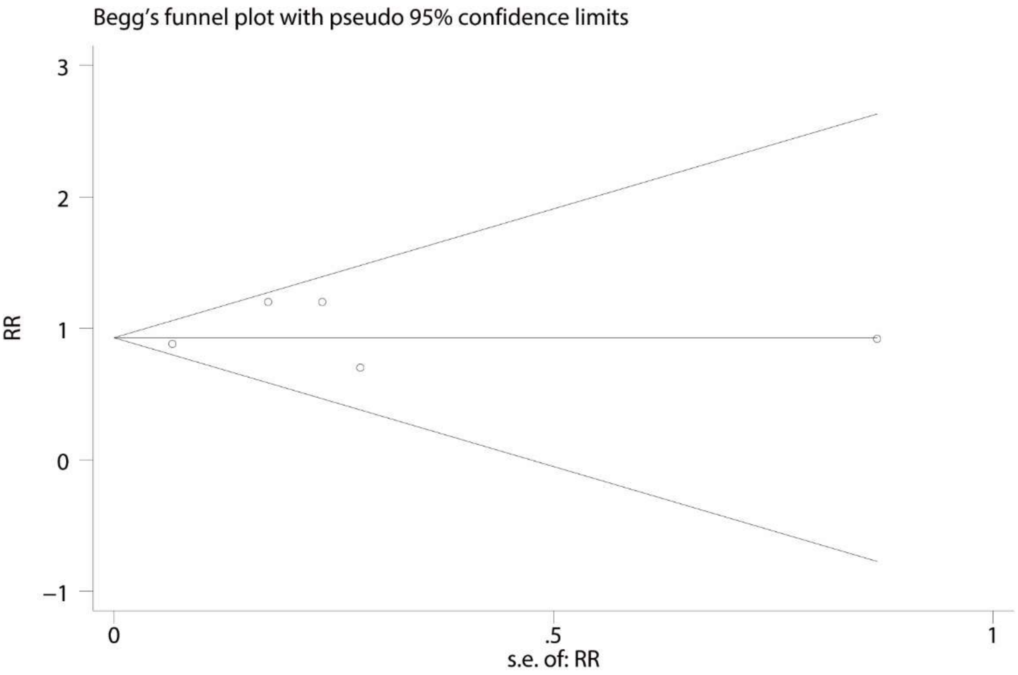

Table S1. Methodological quality of the case-control studies, based on the NOS for assessing the quality of epidemiological studies.

				**Selection**		**Comparability**		**Exposure**			
Study	Year	Definition of cases	Represent ativeness of cases	Selection of controls	Definition of controls	Important Factor	Additional factor	Ascertain ment	Same method for subjects	Non-respo nse rate*	Totle score
Liu B	2019	1	1	1	0	0	1	0	1	0	5
Lin YS	2017	1	1	1	0	1	1	0	1	1	7
Mahale P	2017	1	1	1	1	1	1	0	1	1	8
Kamiza AB	2016	1	1	1	0	1	0	0	1	1	6

Table S2. Methodological quality of the cohort studies, based on the NOS for assessing the quality of epidemiological studies.

		Selection				Comparability		Outcome			
Study	Year	Representativeness of exposed cohort	Representativeness of unexposed cohort	Ascertainment of exposure	Outcome was not present at start	Important Factor	Additional factor	Assessment	Exposure Follow-up long enough for outcomes to occur	Adequacy of follow-up	Totle score
Nyberg AH	2019	1	1	1	0	1	1	1	0	1	7
Liu X	2017	1	1	1	1	0	0	0	1	1	6
Allison RD	2015	1	1	1	1	1	1	1	0	1	8
Hofmann JN	2011	1	1	0	0	0	1	1	1	1	6
Omland LH	2010	1	1	1	1	1	0	0	1	1	7
Gordon SC	2010	1	1	1	1	0	1	1	1	1	8
Amin J	2006	1	1	1	1	0	1	1	1	1	8
